# Origins of Highly Stable Al-evaporated Solution-processed ZnO Thin Film Transistors: Insights from Low Frequency and Random Telegraph Signal Noise

**DOI:** 10.1038/srep16123

**Published:** 2015-11-03

**Authors:** Joo Hyung Kim, Tae Sung Kang, Jung Yup Yang, Jin Pyo Hong

**Affiliations:** 1 Department of Physics, Hanyang University, Seoul, 133-791, Korea; 2 Photovoltaic Development Team, Samsung SDI, Cheonan-si, Chungcheongnam-do, 331-710, Korea

## Abstract

One long-standing goal in the emerging field of flexible and transparent electronic devices is to meet the demand of key markets, such as enhanced output performance for metal oxide semiconductor thin film transistors (TFTs) prepared by a solution process. While solution-based fabrication techniques are cost-effective and ensure large-area coverage at low temperature, their utilization has the disadvantage of introducing large trap states into TFTs. Such states, the formation of which is induced by intrinsic defects initially produced during preparation, have a significant impact on electrical performance. Therefore, the ability to enhance the electrical characteristics of solution-processed TFTs, along with attaining a firm understanding of their physical nature, remains a key step towards extending their use. In this study, measurements of low-frequency noise and random telegraph signal noise are employed as generic alternative tools to examine the origins of enhanced output performance for solution-processed ZnO TFTs through the control of defect sites by Al evaporation.

A metal oxide semiconductor-based thin film transistors (TFTs) have been the focus of immense interest as one of the most reliable building blocks to meet the demand of large area display industries. Among the most prominent advantages of TFTs are their high electrical mobility, good transparency in the visible light regime, and high on/off ratio^1^,^2^,^3^,^4^. To ensure such features, a variety of vacuum deposition methods have been employed to manufacture TFTs, including radio frequency (RF) sputtering, pulsed laser deposition, and atomic layer deposition^1^,^2^,^3^,^4^. In recent years, solution-processed TFTs have emerged as a promising alternative for use in simple fabrication procedures, thereby enabling the production of low-cost electronics. Furthermore, solution methods can be carried out at low temperatures (<300 ^°^C) and allow for easy control of various dopants so as to facilitate the development of various metal-doped ZnO TFTs^5^,^6^. However, the widespread use of solution-based techniques remains a challenge due to the presence of unintended defects introduced during growth. For example, a thin active layer (<10 nm) can be created during growth due to high volatility, rapid decomposition, and concentration limitations. This layer serves to provide inherent spatial charge traps at the interface and surface of the device^7^,^8^,^9^. The formation of spatial traps acting as defect states is also induced by inherently vulnerable metal-oxygen bonding structures^10^. Therefore, intentional control over the concentration of spatial trap states is one of the most long-standing goals in the development of cost-effective TFTs.

To date, numerous reports have been published on the successful enhancement of output performance in solution-processed metal oxide semiconductor TFTs^11^,^12^,^13^. Our previous studies also addressed the concept of Al-evaporated ZnO TFTs exhibiting highlystable device performance in air^14^,^15^,^16^. Control of the carrier concentration and defect states by varying the time of Al evaporation onto a thin ZnO layer had a significant effect on device performance. However, the photoluminescence [PL] spectrum measurements (not shown in this figure) exhibited no large difference in the deep level statesafter various Al incorporation, except a slight peak shift in the near band edge spectrum depending on Al evaporation time. In particular, an enhancement in electrical features was likely to arise from a both a reduction in weak Zn–O bonding, whichis related to shallow donor trap states through Zn substitution by Al, and a decrease in adsorbed oxygen, which is related to the deep level trapping of electrons through the passivation role of Al-evaporated ZnO TFTs^14^,^15^. However, questions still remain regarding how carriers are transported between trap sites under different biases and what the main conduction features are after Al evaporation.

In an attempt to answer the aforementioned questions, low frequency noise (LFN) and random telegraph signal noise (RTN) analyses were utilized as alternative techniques to justify the role of Al evaporation on the enhanced electrical features discussed in previously published papers^14^,^15^. In general, the trapping of carriers within the bandgap of a semiconductor severely limits the performance of analog and digital devices. Therefore, noise characteristics that are governed by the trapping and de-trapping of carriers have garnered great interest in efforts to understand degradation and functionality in semiconductor devices^17^,^18^,^19^,^20^. In this work, observed LFN behaviors were attributed to traps within the bandgap of metal oxide semiconductors and two possible fluctuation sources: carrier number fluctuation (CF) and mobility fluctuation (MF). The number of CFs and MFs that play an important role in noise features were considered in order to verify the experimental findings at different sample and bias conditions^17^,^18^,^19^,^20^,^21^,^22^.

This letter addresses the origins of the LFN and RTN observed in various solution-processed ZnO TFTs with and without Al evaporation on the back channel of the ZnO active layer. The RTN responses were tested at each point of the drain currents, thereby providing two discrete switching events between two levels (high-and low-current states). Such events are induced by carrier capture and emission events, depending on the gate voltage. Plots of the drain current power spectral density versus the drain current of proper Al-evaporated ZnO TFTs confirmed a transition between CF and MF models in the vicinity of the threshold voltage. Based on the observed RTN features, we describe a possible mechanism for stable device performance after Al evaporation that is mainly associated with carrier trap and de-trap behaviors at trap centers in different gate voltage regimes.

## Results

### I-V response and typical low-frequency noise behaviors for ZnO-based TFTs

**Figure 1:**
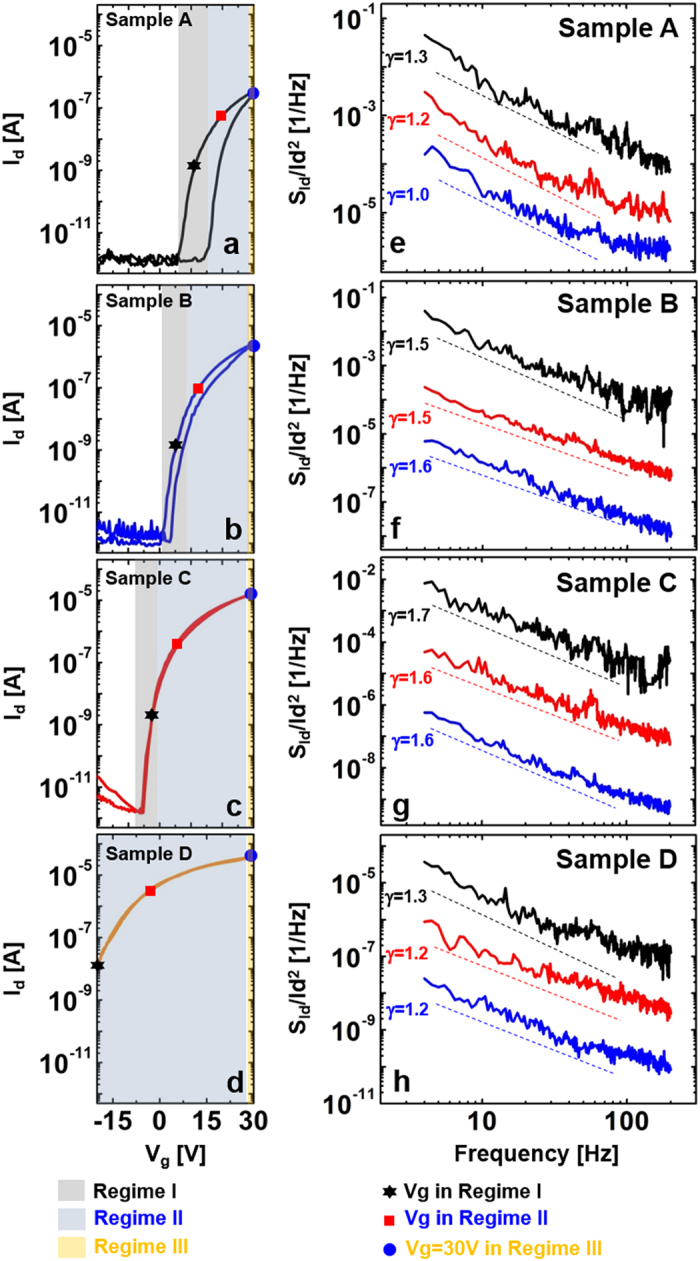
Transfer I-V curves and drain current normalized noise spectral densities (S_Id_/I_d_^2^) for pure ZnO [Sample A] and Al-evaporated ZnO TFTs [Samples B, C, and D]. The S_Id_/I_d_^2^ versus frequency plots for each sample were recorded at a fixed drain-to-source voltage (V_d_ = 10 V). Three points (black stars, red squares, and blue circles in the I-V curve) were chosen for the S_Id_/I_d_^2^ measurements. The channel width (W) and length (L) were 500 μm and 50 μm, respectively. (**a**) I-V response of Sample A without Al evaporation on the back channel of the ZnO layer. (**b**–**d**) I-V responses of Samples B, C, and D, respectively. Transfer electrical responses of Samples B, C, and D reveal negative V_th_ shifts when compared to Sample A. Highly stable and enhanced electrical behaviors with a high on/off ratio were observed with a 20 s Al evaporation time [Sample C]. (**e**) S_Id_/I_d_^2^ plot for Sample A, where each colored line represents the S_Id_/I_d_^2^ curve measured at different gate voltages. (**f**–**h**) S_Id_/I_d_^2^ plots for Samples B, C, and D, respectively. The S_Id_/I_d_^2^ values decrease with increasing gate voltage. All samples show a 1/f^γ^ dependency with γ = 1.2 ~ 1.7, indicating that all of the ZnO TFTs exhibit typical low frequency noise characteristics.

Figure 1 shows the transfer I-V curves and drain current normalized noise power spectral densities (S_Id_/I_d_^2^) of pure ZnO [Sample A] and Al-evaporated ZnO TFTs [Samples B, C, and D] measured over three different voltage regimes; the channel width (W) and length (L) were 500 μm and 50 μm, respectively. For convenience, the three gate voltage (V_g_) regimes are denoted as regime I (in the vicinity of the threshold voltage), regime II (above the threshold voltage), and regime III (far away from the threshold voltage; in this letter, V_g_ = 30 V). The Al evaporation times for Samples B, C, and D of Fig. 1 were 10, 20, and 40 s, respectively. More detailed noise power spectral density (S_Id_) versus frequency data for Samples A-D are given in Figure S1 of the Supporting Information. The representative transfer I-V curves for Samples A, B, C, and D are displayed in Fig. 1a–d, respectively; all samples exhibit typical *n*-channel behavior. It is widely believed that the hysteresis window feature in the I-V curves represents the deep level trapping of electrons with a long release time, whereas the mobility is primarily determined by shallow trap states with a short release time^23^. The I_d_ values in all regimes increase with longer Al evaporation times. Sample A exhibited a large hysteresis feature, while the current performance of Sample C rapidly improved without hysteresis loss. The enhancement in current performance for Sample C can be attributed to a decrease in oxygen vacancy-related porous sites, which in turn leads to a reduction in the defect density as previously reported by other groups^15^,^16^. The observed I-V curves were separated into regions in the vicinity of the threshold regime (gray color) and above the threshold regime (blue color), depending on the drain current. Shown in Fig. 1e–h are the drain current normalized power spectral density (S_Id_/I_d_^2^) plots used to observe LFN features for Samples A, B, C, and D, respectively. The S_Id_/I_d_^2^ versus frequency (range = 4 Hz to 200 Hz) plots were recorded at a fixed drain-to-source voltage (V_d_ = 10 V). Three points (black stars, red squares, and blue circles in the I-V curves of Fig 1a–d) were chosen for the S_Id_/I_d_^2^ measurements. As evident in Fig. 1e–h, S_Id_/I_d_^2^ decreases with increasing drain currents for all samples, reflecting typical LFN features observed for conventional metal oxide semiconductor field effect transistors (MOSFET)^17^,^18^,^19^,^20^,^21^,^22^. Thus, the LFN characteristics of our ZnO TFTs may be expressed by well-known LFN models frequently used for conventional MOSFET devices (this topic will be addressed later). In addition, S_Id_/I_d_^2^ exhibited a frequency dependency with a slope of 1/f ^γ^ slope (γ = 1.2 ~ 1.7) for all samples. The similarity of the noise slopes implies that the nature of noise in all specimens is also similar, i.e., carriers are trapped and de-trapped at defect centers. When examining the results obtained for Samples A, B, and C, it can be seen that increasing the Al evaporation time served to increase γ. For example, the value of γ (1.6 ~ 1.7) for Sample C with stable I-V characteristics was larger than that of Sample A (1.0 ~ 1.3) with unstable I-V features. The larger γ value for Sample C may suggest that the fluctuation of carriers with long lifetimes has a more significant impact on the LFN behavior when compared to that of carriers with a relatively short lifetime. However, the value of γ (1.2 ~ 1.3) for Sample D was similar to that of Sample A, which means that the fluctuation of carriers with both long and short lifetimes contributes to the LFN characteristics over the entire frequency range. As such, more empirical results and comparisons are needed to establish a clearer empirical model or explanation for the origin of the various γ values observed in the samples.

### LFN responses and possible natures of pure ZnO and Al-evaporated ZnO TFTs

**Figure 2:**
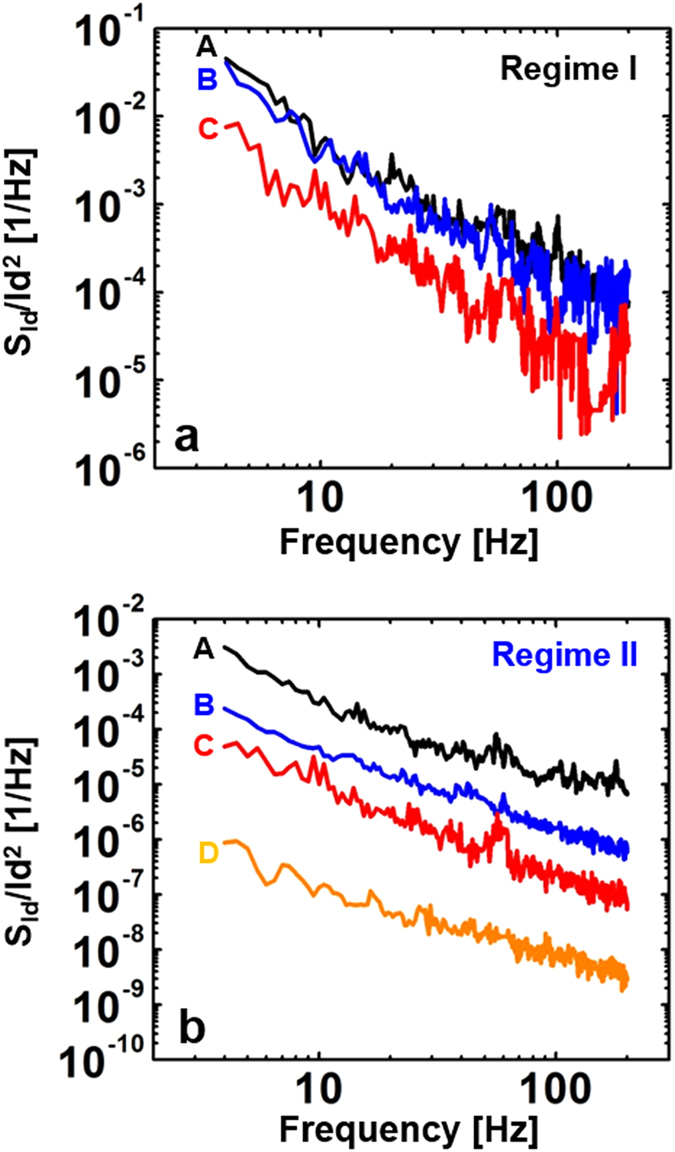
Comparison of normalized noise spectral density (S_Id_/I_d_^2^) plots for Samples A, B, C, and D over two different regimes. (**a**) S_Id_/I_d_^2^ plots measured in regime I; Sample D was excluded due to its high leakage response in regime I. (**b**) S_Id_/I_d_^2^ plots acquired in regime II. Note that proper Al evaporation enhances the noise characteristics in all bias regimes, possibly due to a reduction in the number of traps.

To verify the differences in noise characteristics for Samples A, B, C, and D, the S_Id_/Id^2^ responses in regimes I and II of Fig.1e–h were re-plotted. Figure 2 shows the S_Id_/Id^2^ curves corresponding to Samples A (black lines), B (blue lines), and C (red lines) in regimes I (Fig. 2a) and II (Fig. 2b); Sample D was excluded due to its high leakage response in regime I of Fig. 1d. As displayed in Fig. 2a,b, the noise level of Sample A was always higher than that of Samples B, C, and D in all regimes. Such results indicate an enhancement in noise characteristics upon Al evaporation onto the ZnO layer, possibly due to a reduction in carrier trap or scattering centers that act as the main LNF sources. In addition, a significant decrease, by a factor of 10^4^, in the magnitude of noise was observed for Sample D when compared to that of Sample A (see Fig. 2b). This also suggests a reduction in noise sources for the ZnO TFTs with longer Al evaporation times.

To further clarify the above observations regarding the effect of Al evaporation, two specific noise models frequently implemented for field effect transistors (FET) were adopted. First, the CF model describes fluctuations in the carrier density due to electrons localized at trap sites near the channel–oxide interface or other deep level traps induced by weak structural bonding. Second, the MF model describes fluctuations in charge mobility due to fluctuations in the mean free path of electrons. According to the CF model^17^, S_Id_ in regime I can be expressed as


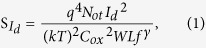


where q is the elementary charge, k is the Boltzmann constant, T is temperature, Not is the trap density per unit area, C_ox_ is the gate capacitance per unit area, and γ is the slope parameter. Furthermore, the value of SId above the threshold region (regimes II and III) can be expressed as^17^


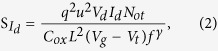


where μ is the charge carrier mobility. According to the Equation 1 and 2, the S_Id_ – I_d_ dependence gives two different slopes, 2 or 1, for regimes I and II/III, respectively. According to the MF model based on Hooge’s empirical law^17^, S_Id_ can be expressed as


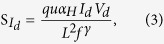


where α_H_ is the Hooge parameter that allows for a comparison of noise characteristics in different conductive materials. Equation 3 suggests a slope of 1 from the S_Id_ – I_d_ plot, which is similar to that derived from Equation 2.

**Figure 3:**
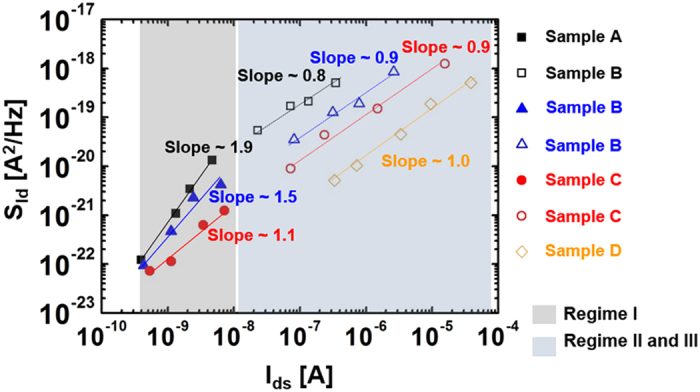
Drain current power spectral density (S_Id_) versus drain current for Samples A, B, C, and D. The S_Id_ plots as a function of drain current were recorded at ƒ = 50 Hz and V_d_ = 10 V. In regime I (grey color), the slopes vary from 1.9 to 1.1. The slope of Sample A is about 2 times larger than that of Sample C in regime I, while the slopes of all samples are approximately equal to 1 in the other regimes. Sample D was excluded due to its high leakage response in regime I.

To formulate a relationship between the two noise models and the observed results, plots of SId versus drain current (I_d_) were generated for each sample over all regimes; the results are shown in Fig. 3. The SId plots as a function of drain current were recorded at f = 50 Hz and V_d_ = 10 V. In regime I (gray color in Fig. 3), the slope was found to vary from 1.9 to 1.1 depending on the sample (Sample D was excluded due to its high leakage response in regime I). More detailed S_Id_ – I_d_ data recorded at different frequencies for Samples A-D are presented in Figure S2 of the Supporting Information. The slope of Sample A was about 2 times larger than that of Sample C in regime I, while the slopes of all samples in regime II and III (blue color in Fig. 3) were approximately equal to 1. Thus, the slopes of all specimens may be well-described by fitting the models to the SId plots over all regimes. For example, Sample A shows nearly quadratic variations as a function of the drain current Id with a slope close to 1.9 in regime I. This implies that the notion of the electrons drifting toward the drain side is appropriate for the CF model expected from Equation 1. That is, the LFN characteristics of Sample A are a result of carrier trapping and de-trapping by trap centers in regime I. The slopes obtained for all samples in regime I progressively vary from 2 to 1 with increasing Al evaporation time. Such a variation in slope indicates a rapid reduction in the density of traps inside the active region with Al evaporation. This in turn leads to change from CF in Equation 1 to MF in Equation 3. However, the current fluctuation of Sample B with a slope ~ 1.5 is described by almost equal proportions of both the CF and MF models. In regimes II and III, all SId values have a linear relationship (slope ~ 1) with Id. This relationship is in good agreement with the fitting results obtained from Equation 2 and 3. With the exception of a buried channel far away from the gate insulator, typical FETs allow for an effective channel region under the gate insulator interface that is associated with the trap density in the above threshold regime^24^,^25^. In our work, the ZnO TFTs permit the presence of an effective channel region placed close to the SiO_2_/ZnO interface due to the formation of thin ZnO active layer (<10 nm). Therefore, the dominant LFN characteristics in regimes II and III can be described by the CF model. As expected from Equation 2 and the empirical results of Fig. 3, a reduction in the trap density upon Al evaporation corresponds to the LFN behaviors observed for Samples B, C and D.

**Figure 4:**
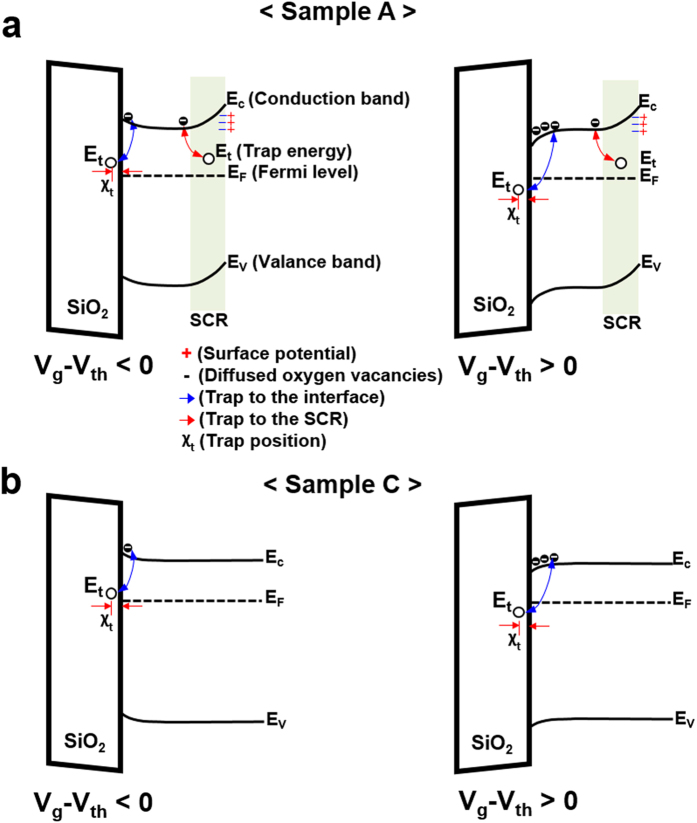
Gate voltage-dependent band diagrams for depicting the noise behaviors of Samples A and C. (**a**) Band diagrams of Sample A in the V_g_ – V_th_ < 0 (left) and V_g_ – V_th_ > 0 (right) regimes. In sample A, traps at the SCR (which is created by attracted O^2−^ ions) and SiO_2_/ZnO interface may contribute to the noise characteristics in all regimes. Thus, two trap energies (E_t_) are placed at each trap center. (**b**) Band diagrams of Sample C in the V_g_ – V_th_ < 0 (left) and V_g_ – V_th_ > 0 (right) regimes. In Sample C, the noise behavior mainly arises from the SiO_2_/ZnO interface.

To further illustrate the observed LFN behaviors, simple gate voltage-dependent band diagrams were constructed. Shown in Fig. 4a,b are band diagrams for Samples A and C, respectively, at two different gate bias regimes. The left and right sides of each figure represent the band diagrams when V_g_ – V_th_ < 0 and V_g_ – V_th_ > 0, respectively. (Where V_th_ is the gate threshold voltage.) As displayed in Fig. 4a, Sample A contains a space charge region (SCR, denoted in blue) that is initially present during solution-processed growth. Large oxygen vacancies working as donors diffuse out toward the back channel surface (BCS) due to a surface potential energy generated by O^2−^ ions attracted from the exposed outside surface. This results in conduction band (E_c_) and valence band (E_v_) bending far from the Fermi level (EF), as depicted in Fig. 4a. The SCR includes weak Zn-O bonding as well as pore traps in the area close to depletion region, thereby establishing a narrow effective channel region under the SiO_2_ gate layer due to the enhanced surface potential effect. Therefore, the LFN features of Sample A arise from weak Zn-O bonding and pore traps at the BCS, as well as from trap centers at the SiO_2_/ZnO interface. In contrast, Al evaporation can lead to the removal of weak Zn-O bonds by creating strong Al–O bonding in the SCR region, as shown in Fig. 4b. This in turn reduces the concentration of both weak Zn-O bonds and pore defect states. The result is the presence of a wider effective channel region in Sample C when compared to that of Sample A due to a reduced surface potential effect. Our previous observations gleaned from optical and structural measurements provided evidence for the replacement of weakly bonded Zn-O near the surface with Al-O bonding after Al evaporation on the ZnO active layer^15^. Thus, contributions to the LFN characteristics after Al evaporation mainly come from trap centers at the SiO_2_/ZnO interface.

### RTN analysis in the vicinity of the threshold voltage

**Figure 5:**
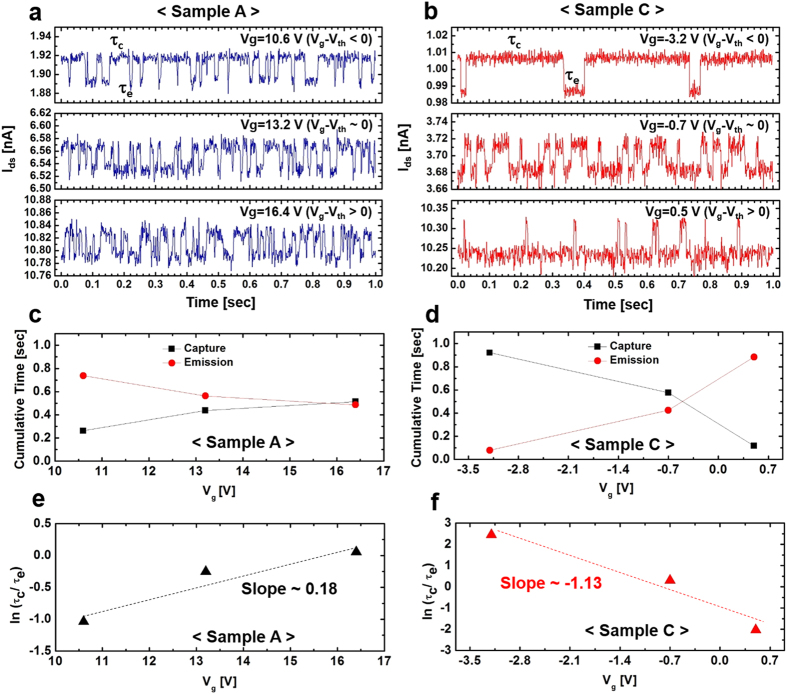
Time domain RTN traces for Samples A and C. The gate voltages in the vicinity of regime I were selected at V_d_ = 10 V for the RTN measurements. Plots of the drain current versus time domain for (**a**) Sample A and (**b**) Sample C. Here, τ_e_ and τ_c_ denote the times to emit and capture carriers in the RTN measurements, respectively. (**c**) Gate voltage-dependent cumulative time plot for electron capture (black squares)/emission (red circles) in Sample A. (**d**) Gate voltage-dependent cumulative time plot for electron capture (black squares)/emission (red circles) in Sample C. Gate voltage-dependent ln τ_c_/ τ_e_ plots calculated for (**e**) Sample A and (**f**) Sample C. Sample A shows a dominant electron emission process in the V_g_ – V_th_ < 0 region, while Sample C displays two distinct gate voltage regions: a V_g_ – V_th_ < 0 region where electron capture is a prime factor, and a V_g_ – V_th_ > 0 region where electron emission is dominant. Sample C shows trends similar to those observed in typical MOSFETs.

To further exploit the nature of the observed LFN sources as proof for the possible physical model proposed above, time-consuming RTN analyses of a time domain plot of I_d_ were conducted; the results are given in Fig. 5. The RTN signals are commonly caused by specific carrier trapping and de-trapping processes that mainly occur at the channel oxide/SiO_2_ interfaces, particularly in small-scale FET devices. The average carrier capture (referred to as a de-trapping process) or emission (referred to as a trapping process) fluctuations are well-described based on the Shockley-Read-Hall (SRH) theory^26^. The carrier emission and capture responsible for RTN take place through a tunneling process from or towards the channel region with specific time constants (τ_c_ and τ_e_) serving as typical RTN parameters. Here, τ_c_ and τ_e_ refer to the carrier capture and emission times, respectively. These times are determined by a separating algorithm from histograms extracted from time domain plots. According to the generally accepted SRH theory, the ratio between τ_c_ and τ_e_ is expressed as


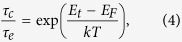


where E_t_ is the trap energy and E_F_ is the Fermi Level. Equation (4) can be used to identify the trap position or trap depth (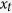) in the gate oxide at some distance from the gate insulator interface as follows^26^


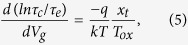


where T_ox_ is the thickness of the gate oxide and 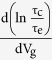 is the slope of a gate-voltage dependence plot.

Shown in Fig. 5a,b are plots of the RTN responses for Samples A and C, respectively. Using a small active area (W = 10 μm and L = 1 μm), three gate voltages (V_g_ – V_th_ < 0, V_g_ – V_th_ ~ 0, and V_g_ – V_th_ < 0) in regime I were selected at V_d_ = 10 V for the RTN measurements. In general, the value of I_d_ in regime I was too high for observations of the RTN response. As evident in Fig. 5a,b, all time domain traces in the three V_g_ regions show distinct high state (capture, τ_c_) and low state (emission, τ_e_) drain currents^26^,^27^. Displayed in Fig. 5c,d are the V_g_ dependences of τ_c_ (black squares) and τ_e_ (red squares) extracted from histograms obtained from Fig. 5a,b, respectively. More detailed histograms for Samples A and C are given in Figure S3 of the Supporting Information. At first, the RTN of Sample C (Fig. 5d) can be described in two distinct gate voltage regions: the V_g_ – V_th_ < 0 regime, where τ_c_ is a prime factor, and the V_g_ – V_th_ > 0 region, where τ_e_ is dominant. However, in Sample A (Fig. 5c) τ_e_ was dominant in the V_g_ – V_th_ < 0 region and both τ_c_ and τ_e_ exhibited almost equal contributions in the V_g_ – V_th_ > 0 regime. That is, Sample C exhibits RTN behaviors that are typical of MOSFETs, while Sample A does not follow such trends. Therefore, it is believed that additional contributions to noise are present in Sample A (as described earlier). The ratios of τ_c_ to τ_e_ for Samples A and C as a function of gate voltage are shown in Fig. 5e,f, respectively. Positive and negative slopes were obtained for Samples A and C, respectively. Furthermore, *x*_*t*_ for Sample C was about 5.9 nm from the SiO_2_/ZnO interface when the contribution to noise from the oxide/SiO_2_ interface only is considered. However, *x*_*t*_ for Sample A was about −0.96 nm with respect to the SiO_2_/ZnO interface. According to Equation 5, such a result does not represent a typical trap depth. That is, a simple analysis of Sample A yields information on the presence of a relatively large number of trap centers in the SCR. These trap centers mainly contribute to the RTN features, as indicated from the LFN characteristics in Figs 2 and 3. In addition, the negative slope of Sample C supports the usual trend of noise sources created by interfaces only, while the presence of a positive slope for Sample A provides experimental evidence for additional noise contributions arising from the SCR. Therefore, we believe that the above experimental findings clearly demonstrate the role of Al evaporation in reducing trap centers that are initially present during the preparation of solution-processed ZnO TFTs.

In summary, the origins of LFN and time-domain RTN features were explored in solution-processed ZnO TFTs with or without Al evaporation on the back channel of the ZnO active layer. The noise associated with Sample A (no Al evaporation) arises from typical trap centers due to unstable bonding at the SiO_2_/ZnO interface as well as from weak Zn-O bonding and pore traps in a space charge region as a result of O^2−^ ion absorption into the back channel surface of the ZnO layer. The LFN of Sample A is mainly determined by carrier fluctuations in all regimes, and the RTN response shows more dominant carrier emission (trap) time counting than capture (de-trap) time counting in the threshold voltage regime. In contrast, the enhanced noise features of Sample C may be ascribed to a reduction in weak Zn–O bonding through Zn substitution by Al and a decrease in adsorbed oxygen via passivation in the Al-evaporated ZnO TFTs. The LFN of Sample C is a mobility noise in regime I, and the RTN characteristics exhibit behavior typically observed in conventional Si-MOSFETs, where only the interface noise contribution is considered. Thus, we anticipate that the ability to control and improve the noise features of TFTs by proper Al evaporation will lead to practical applications for solution-processed oxide semiconductors.

## Methods

### Measurement

Transistor current-voltage characteristics were measured using a HP4156 semiconductor parameter analyzer. Forward and reverse gate biases of the hysteresis were analyzed between −20 and 30 V (at V_d_ = 10 V) in order to generate typical transfer curves. LFN and RTN tests were conducted in a shielded probe-station with the aid of an HP4156 semiconductor parameter analyzer, a BTA 9812B low noise amplifier (for LFN), a SR570 low noise amplifier (for RTN), and an HP 35670A dynamic signal analyzer.

### Fabrication

A ZnO solution was prepared by dissolving 0.005 moles of zinc oxide (Sigma Aldrich 99.999%) in 6 mL of ammonium hydroxide (aqueous solution, Alfa Aesar, 99.9%). To increase the solubility of the ammonia water, the precursor solution of ZnO was kept in a refrigerator for 10 h. After dissolution of the precipitated ZnO particles, the ZnO solution was deposited onto heavily Boron-doped p-type Si with a thermally grown SiO_2_ substrate (200 nm, C_ox_ = 17.25 nF cm^−2^) by spin-coating for 60 s at 5000 rpm. The resulting film with a thickness of 7–8 nm was subsequently annealed at 300 °C for 1 h. Before the deposition process, the SiO_2_/Si substrate was cleaned in a piranha solution (H_2_O_2_:H_2_SO_4_ = 1:1) and then submerged in diluted HF for 1 min so as to remove residual sulfur.

An Al pellet was thermally evaporated under high vacuum (10^−7^ Torr) onto the ZnO films at a deposition rate of 0.1 Å/sec. Upon evaporating the Al layers, a UVC (wave length: 100–280 nm) irradiation process (to promote oxidation of Al) was performed for 5 min, followed by thermal annealing at 100 °C for 15 min. Finally, 100 nm-thick Al electrodes were deposited on the ZnO film via thermal evaporation through a shadow mask. The channel width (W)/length (L) was 500 μm/50 μm for I-V and LFN analysis, and 10 μm/1 μm for determining RTN characteristics.

## Additional Information

**How to cite this article**: Kim, J. H. *et al.* Origins of Highly Stable Al-evaporated Solution-processed ZnO Thin Film Transistors: Insights from Low Frequency and Random Telegraph Signal Noise. *Sci. Rep.* **5**, 16123; doi: 10.1038/srep16123 (2015).

## Supplementary Material

Supplementary Information
