# Supplementary material for: Origins of Highly Stable Al-evaporated Solution-processed ZnO Thin Film Transistors: Insights from Low Frequency and Random Telegraph Signal Noise
Source: Sci Rep. 2015 Nov 3;5:16123. doi: 10.1038/srep16123 (PMC4630786; doi:10.1038/srep16123)
Supplement: Supplementary Information [file srep16123-s1.doc]

**Supplementary Information for “Origins of Highly Stable Al-evaporated Solution-processed ZnO Thin Film Transistors: Insights from Low Frequency and Random Telegraph Signal Noise”**

Joo Hyung Kim^1^, Tae Sung Kang^1^, Jung Yup Yang^2^, and Jin Pyo Hong^1★^

*^1^Department of Physics, Hanyang University, Seoul, 133-791, Korea*

*^2^Photovoltaic Development Team, Samsung SDI,*

*Cheonan-si, Chungcheongnam-do, 331-710, Korea*

**Corresponding author E-mail:** [**jphong@hanyang.ac.kr**](mailto:jphong@hanyang.ac.kr)

Supplementary figures

1. S_Id_ plots for pure ZnO and Al-evaporated ZnO TFTs in regimes I, II, and III.


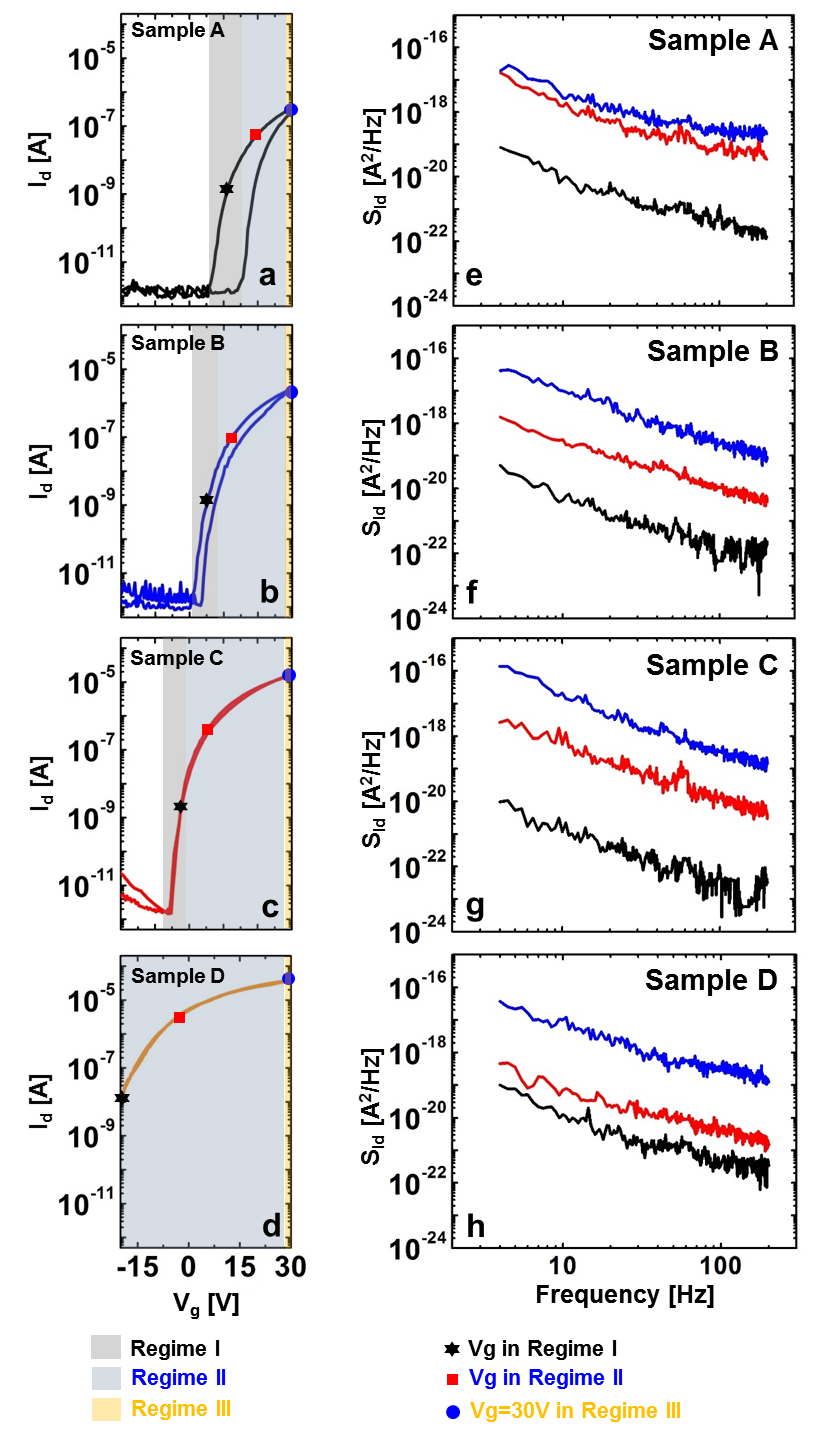


Figure S1. Transfer I-V curves and noise spectral density (S_Id_) versus frequency. (a) I-V response of Sample A without Al evaporation on the back channel of the ZnO layer. (b), (c), and (d) I-V responses of Samples B, C, and D, respectively, after different Al evaporation times (10, 20, and 40 s for Samples B, C, and D, respectively). (e) S_Id_ plot for Sample A, where each colored line refers to the S_Id_ curve measured at a different gate voltage. (f), (g), and (h) S_Id_ plots for Samples B, C, and D, respectively. The S_Id_ value acquired at V_g_ =30 V exhibits the highest noise due to the large signal to noise ratio.

Figure S1 shows the transfer I-V curves and original noise spectral densities (S_Id_) versus frequency for Samples A-D in three different voltage regimes; the channel width (W) and length (L) were 500 μm and 50 μm, respectively. The transfer I-V curves and S_Id_ plots for Samples A-D were used for the plots in Figure 1 of the manuscript. As seen in these graphs, the value of S_Id_ in the three selected V_g_ regimes increases with increasing drain current due to the larger signal to noise ratio, indicating that our ZnO TFTs exhibit noise characteristics typically observed for conventional FETs.

2. S_Id_ versus drain current for Samples A, B, C, and D at 10 Hz and 100 Hz.


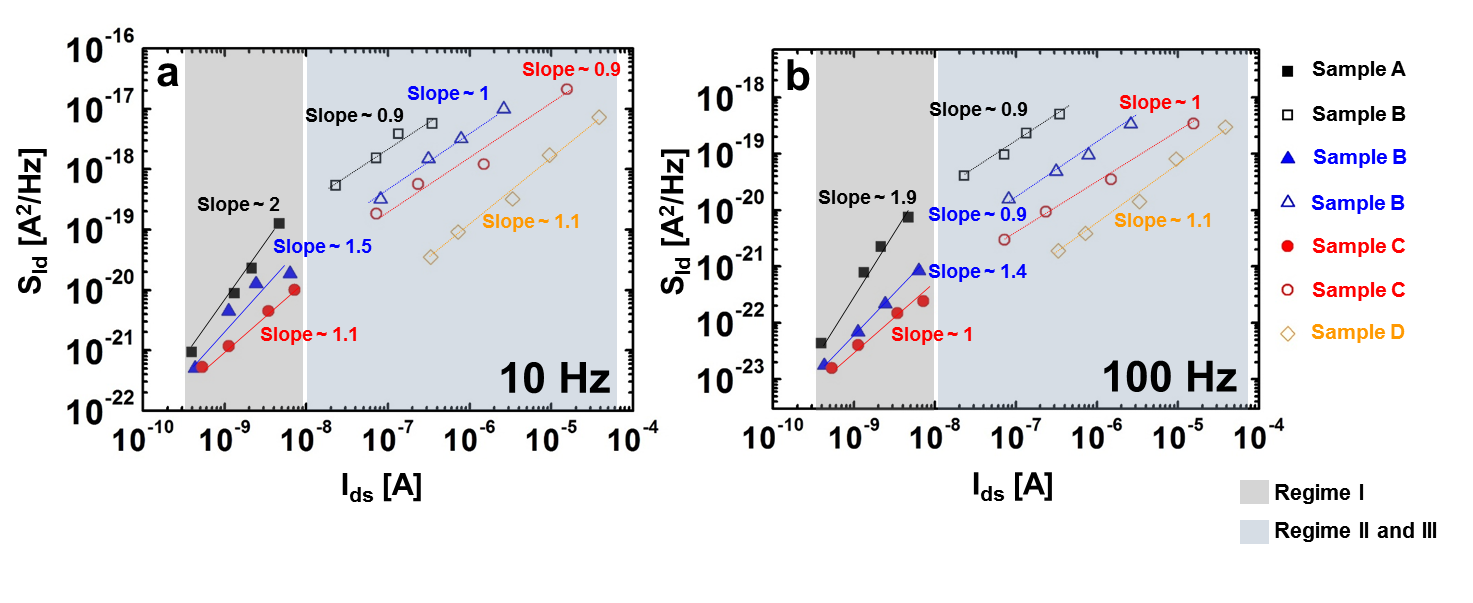


Figure S2. Drain current power spectral density (S_Id_) versus drain current for Samples A, B, C, and D. The S_Id_ plots as a function of drain current were recorded at (a) ƒ=10 Hz and (b) f=100 Hz, with V_d_ =10 V. In regime I of both plots (denoted in gray), a slope variation from 2 to 1 is observed depending on the sample. The slope of Sample A is about 2 times larger than that of Sample C in regime I, while the slopes of all samples are approximately equal to 1 in the other regimes. Sample D was excluded due to its high leakage response in regime I.

Figure S2a and S2b show the S_Id_ – I_d_ curves measured at ƒ= 10 Hz and 100 Hz, respectively. As evident in the plots, the slopes in each regime were almost the same as those measured at f=50 Hz in Figure 3. Thus, observations can be made based on fluctuations in either the mobility or the number of carriers, depending on the sample conditions and regime.

3. Histograms corresponding to the time domain Id plots for Samples A and C


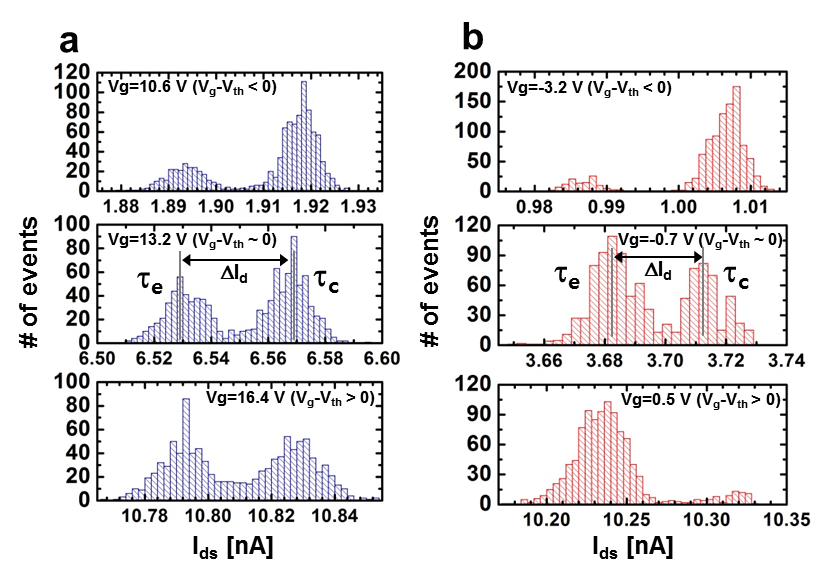


Figure S3. Histogram plots of the time domain RTN for Samples A and C in Figure 5. The number of events for τ_c_ and τ_e_ versus the drain current for (a) Sample A and (b) Sample B. Here, τ_e_ and τ_c_ denote the times to emit and capture carriers in the RTN measurements, respectively. Sample A shows a dominant electron emission process in the V_g_ -V_th_ < 0 region, while Sample C displays two distinct gate voltage regions: a V_g_ -V_th_ < 0 region where electron capture is a prime factor, and a V_g_ -V_th_ > 0 region where electron emission is dominant. Sample C shows a trend similar to that observed in typical MOSFETs.

Figure S3 shows the histograms corresponding to the time domain plots of the drain current for Samples A and C, as represented in Figure 5a and 5b, respectively. The histograms were used to accurately estimate the total capture/emission time and to define ΔI_d_ between the capture and emission times. Displayed in Figure 5c-5f are plots extracted and calculated from the histograms in Figure S3a and S3b.
